# Annual Meeting of the Belgian Society of Radiology (BSR): Programme by the Young Radiologist Section (YRS)

**DOI:** 10.5334/jbsr.1664

**Published:** 2018-11-17

**Authors:** Barbara Geeroms

**Affiliations:** 1 UZ Leuven, BE

It has become a good tradition: the parallel programme organised by the Young Radiologists during the Annual Meeting of the BSR. For the fourth year in a row, the young and eager have invited a nice selection of renowned experts in the fields of Head and Neck Radiology, Interventional Radiology and Artificial Intelligence.

The first part of the morning will cover several radiological challenges in head and neck imaging and will be moderated by Anne-Sophie Vanhoenacker and Barbara Geeroms, both radiology residents at UZ Leuven.

**Figure:**
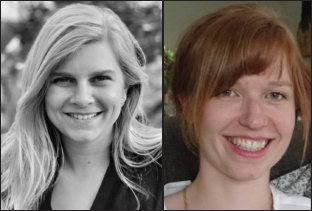
**Dr. Anne-Sophie Vanhoenacker** (left), **Dr. Barbara Geeroms** (right)

**Prof. Marc Lemmerling** will start off with a comprehensive presentation on temporal bone CT. Professor Lemmerling has been a head and neck imaging expert for over twenty years and made a doctoral thesis on ‘HRCT and MR Imaging of the Middle and Inner Ear’ at the University of Ghent. He has been working as a radiologist in AZ Sint-Lucas in Ghent since 2000. No ossicle abnormality escapes the eye of this temporal bone specialist.

**Figure:**
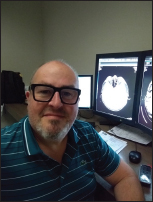
**Prof. Marc Lemmerling**

How do we best handle these cysts and nodules in the thyroid gland that we are often so uncertain about? **Dr. Kunwar Bhatia** will teach us her tips and tricks and more. Just read the Head and Neck editorial of this issue to learn more about Dr. Bhatia.

And finally, when you find yourself facing a difficult head and neck imaging case at the most exotic hours in the emergency department, just think back at the presentation of **Dr. Yannick De Brucker** at this Annual Meeting. Dr. Yannick De Brucker has been a head and neck imaging specialist for several years now, after completing two fellowships in the field: a first at the University of Florida in 2015 and a second at UZ Leuven in 2016. Currently he is working as a staff member in UZ Brussel.

**Figure:**
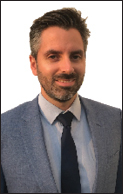
**Dr. Yannick De Brucker**

During the second part of the morning sessions, several experts on wires and tubes will tell you all about how to really get interventional. **Dr. Astrid Van Hoyweghen**, radiologist at UZA, and **Dr. Pierre-Antoine Poncelet**, radiology resident at UCL University Hospital in Brussels, will moderate the lectures.

**Figure:**
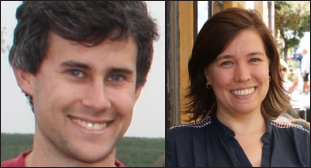
**Dr. Pierre-Antoine Poncelet** (left), **Dr. Astrid Van Hoyweghen** (right)

The first speaker is **Prof. Romanic Loffroy** from the University of Dijon School of Medicine. He was a postdoctoral research fellow at Johns Hopkins Medical Hospital in Baltimore and is currently doing research, among other things, in new interventional radiology therapies for liver cancer, deep vein thrombosis, and arterial diseases and in new embolic materials. He will share all his secrets on biopsies and drainage techniques.

**Figure:**
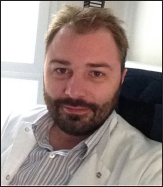
**Prof. Romanic Loffroy**

A dilemma probably every radiologist has faced in the emergency department when he or she has just diagnosed an active hemorrhage: Is this a case for our interventional radiology colleague or do we send this to the surgeons to handle? **Dr. Otto van Delden** will address this and other questions in his talk on the management of hemorrhages. You can read more about Dr. van Delden in the Interventional Radiology editorial.

Finally, **Prof. Marco Midulla** will take us on a short journey through his views on the future of interventional radiology and the emerging techniques in the field. Professor Midulla is currently working at Dijon University Hospital. He made a doctoral thesis on ‘Hemodynamic Aortic Imaging’ and got specific training in vascular and interventional radiology in major aortic centers in Stanford (US), Toulouse, and Lille.

**Figure:**
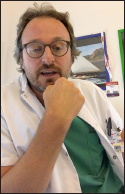
**Prof. Marco Midulla**

Traditionally the afternoon starts off with a message form **Prof. Geert Villeirs**, president of the Belgian Society of Radiology, in which he will discuss actual problems and challenges in radiology, as well as his view on the future of radiology.

**Figure:**
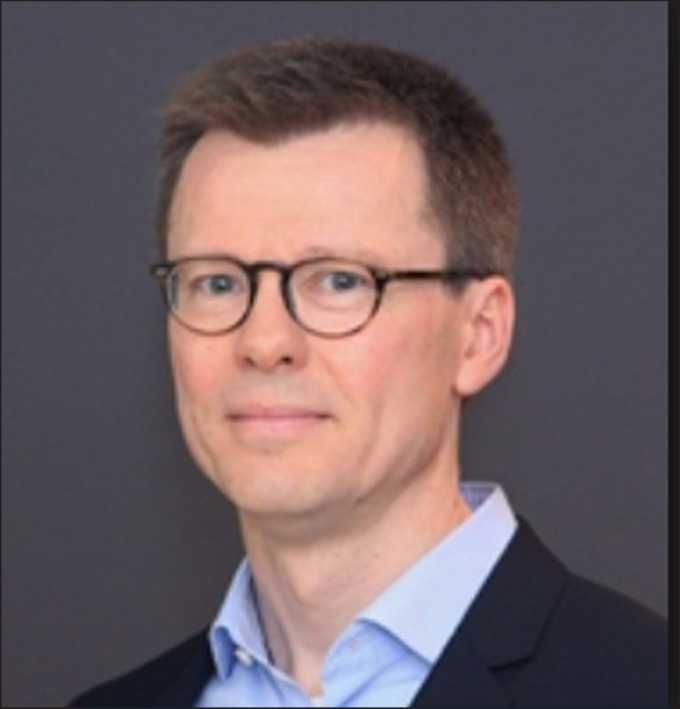
**Prof. Geert Villeirs**, president of the BSR

Following this message, **Dr. Matthias Lavens** and **Dr. Barbara Geeroms**, both radiology residents at UZ Leuven, will share the results of a survey performed by the YRS on actual and future job opportunities in radiology in Belgium.

**Figure:**
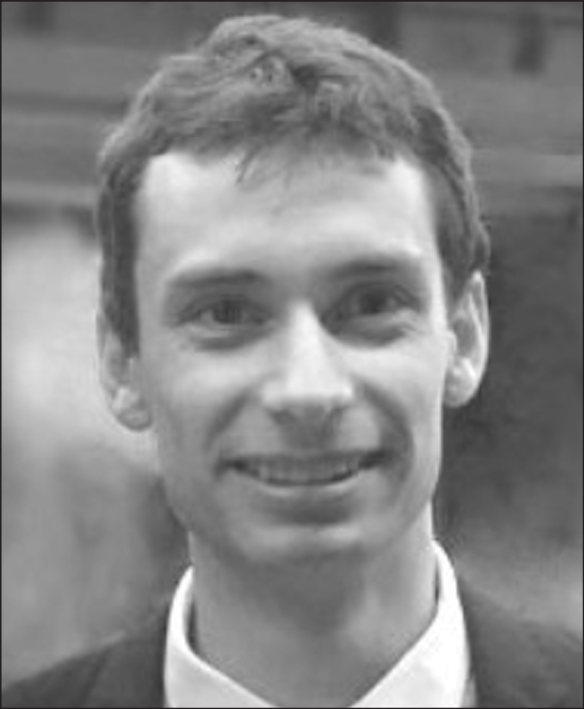
**Dr. Matthias Lavens**

The last sessions of the 2018 Annual Meeting will cover what is probably the hottest topic in radiology these days: artificial intelligence (AI). Moderators **Dr. Cedric Bohyn** and **Dr. Matthieu Deltomme**, both radiology residents at UZ Leuven, will guide us from speaker to speaker.

**Figure:**
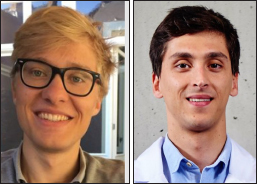
**Dr. Mathieu Deltomme** (left), **Dr. Cedric Bohyn** (right)

The first speaker is **Prof. Erik Ranschaert**, vice-president of EuSoMi. He made a doctoral thesis at the University of Antwerp titled ‘The Impact of Information Technology on Radiology Services’. He is an expert on artificial intelligence. Currently he is working at the ETZ Teaching Hospital in Tillburg, the Netherlands. Professor Ranschaert will share his views on the emergence of artificial intelligence.

**Figure:**
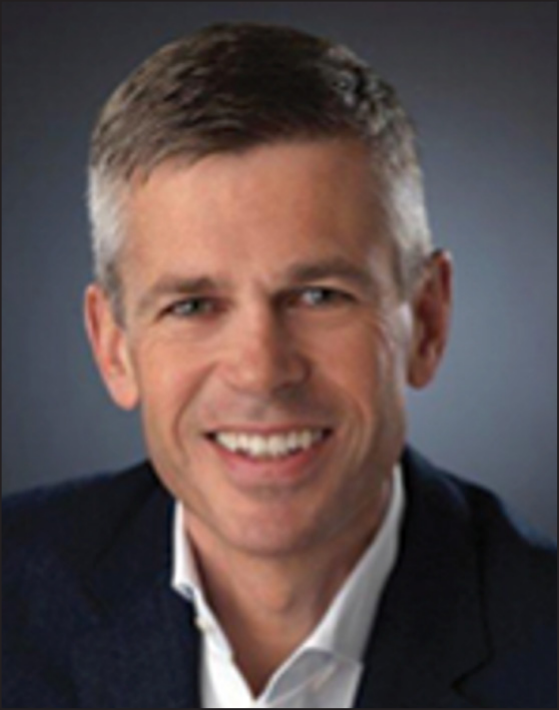
**Prof. Erik Ranschaert**

During the next lecture, two lawyers from law firm ‘**Dewallens & Partners**’ will elaborate on the legal aspects and possible concerns that AI will bring us.

**Dr. iur. Christophe Lemmens** and **Dr. iur. Nils Broeckx** both studied law and obtained a doctoral degree at the University of Antwerp. They are both specialists in digital data management.

**Dr. iur. Christophe Lemmens** has a broad orientation in health law and is specialized in patients’ rights and obligations and in medical liability.

**Dr. iur. Nils Broeckx** has special expertise in medical liability and data protection law (including GDPR) with a specific focus on the health care sector.

**Figure:**
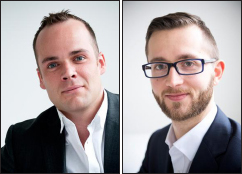
**Christophe Lemmens** (left), **Nils Broeckx** (right)

The final talk of the day will be given by **Prof. Bram van Ginneken** from Radboud University Medical Center, the Netherlands. In 2001 he obtained his PhD at the Image Sciences Institute on Computer-Aided Diagnosis in Chest Radiography. He pioneered the concept of challenges in medical image analysis and is thus the designated person to tell us more about real-life artificial intelligence applications.

**Figure:**
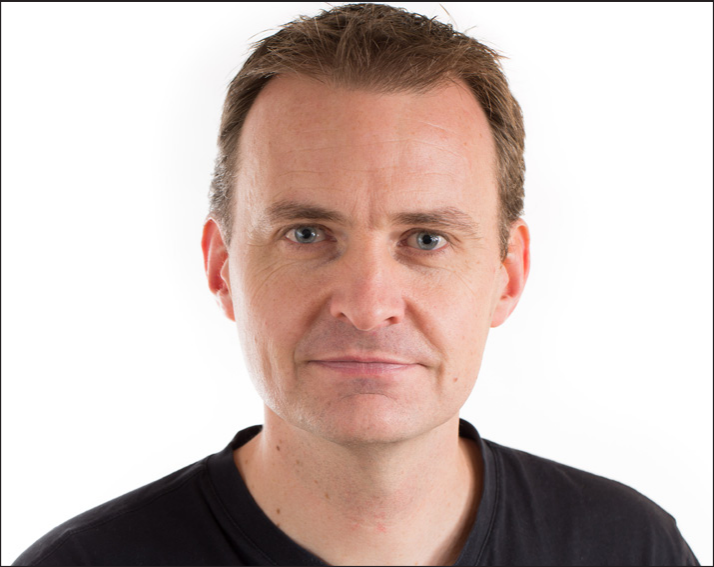
**Prof. Bram van Ginneken**

We hope you will enjoy the programme we have put together for this Annual Meeting. We also have a little ‘surprise act’ in store for you and the lucky hundred who registered in time will be able to enjoy the very first ultrasound party afterwards!

